# Extracellular Vesicles and Pregnancy-Related Hypertensive Disorders: A Descriptive Review on the Possible Implications “From Bench to Bedside”

**DOI:** 10.3390/biology14030240

**Published:** 2025-02-27

**Authors:** Elena Grossini, Daniela Surico, Sakthipriyan Venkatesan, Mohammad Mostafa Ola Pour, Carmen Imma Aquino, Valentino Remorgida

**Affiliations:** 1 Laboratory of Physiology, Department of Translational Medicine, Università del Piemonte Orientale, 28100 Novara, Italy; sakthipriyan.venkatesan@uniupo.it (S.V.); 20046522@studenti.uniupo.it (M.M.O.P.); 2 Gynecology and Obstetrics Unit, Department of Translational Medicine, Università del Piemonte Orientale, 28100 Novara, Italy; daniela.surico@med.uniupo.it (D.S.); c.immaquino@gmail.com (C.I.A.); valentino.remorgida@uniupo.it (V.R.)

**Keywords:** biomarker, pre-eclampsia, prevention, vesicles, pregnancy-related hypertension, circulating markers, obstetric diagnosis

## Abstract

Pregnancy involves extracellular vesicles (EVs) through mechanisms that are poorly understood to date. Furthermore, it is not surprising that EVs may also be involved in the pathophysiology of pre-eclampsia (PE) and gestational hypertension, two clinical conditions with high morbidity and mortality, given their capacity to mediate intracellular communications and regulate inflammation and angiogenesis. We searched major online scientific search engines (PubMed, Google Scholar, Scopus, WES, Embase, etc.) using the terms “Preeclampsia”, “Pregnancy”, “Hypertension”, “Pregnancy-related hypertension”, “Extracellular vesicles”, “Biomarkers”, “Gestation” AND “Obstetrics”. Finding potential early biomarkers of risk or illness progression would be essential for the optimum care of expectant mothers with the aforementioned conditions. Nevertheless, none of the various screening assays that have been discovered recently have shown high predictive values. The analysis of EVs in the peripheral blood starting from the first trimester of pregnancy may hold great promise for the possible correlation with gestational hypertension problems and represent a marker of the early stages of the disease. EVs use may be a novel therapeutic approach for the management of various illnesses, as well. In order to define EVs’ function in the physiopathology of pregnancy-associated hypertension and PE, as well as their potential as early biomarkers and therapeutic tools, we have compiled the most recent data in this review.

## Simple Summary

Pre-eclampsia (PE) is a systemic condition that affects 3 to 5% of pregnant women with new-onset hypertension and proteinuria after 20 weeks of gestation. This syndrome causes maternal and neonatal morbidity and mortality. The term “extracellular vesicles (EVs)” refers to vesicles released by cells and can be divided into exosomes, microvesicles, and apoptotic bodies. EVs have an impact on the physiology of pregnancy and on diseases associated with pregnancy because they can be implicated in the communication between the mother and the fetus. The intricate interactions between placental and maternal cell-derived EVs ought to shed light on the mechanisms behind PE. Developing a panel of biomarkers to identify pregnant women at higher risk of developing PE may be made possible through a better understanding of the functions that EVs play in the pathophysiology of PE.

## 1. Introduction

### 1.1. Pregnancy-Related Hypertensive Disorders

Spiral artery remodelling takes place during pregnancy, as the next paragraphs better explain. The endothelial cells and vascular smooth muscle of the spiral decidua artery are replaced by trophoblastic cells that penetrate the decidua. To increase the partial pressure of oxygen in the placenta and decrease systemic vascular resistance, maternal blood arteries begin to perfuse the interchorionic space [1]. Using the VEGFR-1 receptor, angiogenesis is altered by angiogenic factors, vascular endothelial growth factors (VEGFs), and placental growth factors (PlGFs). In order to prevent allogeneic activities against the fetus, uterine natural killer cells (NK) and regulatory T cells are essential during pregnancy [2,3]. Decidual NK cells use chemokines, interleukin-8, interferon-inducible protein-10, and other angiogenic factors to regulate trophoblast invasion [4].

For reasons that are still unclear, when this system is altered, hypertensive disorders may affect pregnancy. They complicate 10–20% of pregnancies in developed countries and 10% of pregnancies worldwide. Severe pre-eclampsia (PE) and eclampsia are a frequent cause of maternal death (15–20% of cases worldwide and 10% in Italy). Women should be tested for clinical risk of PE from early pregnancy [5].

Hypertension in pregnancy is defined as the detection of diastolic blood pressure values ≥ 90 mmHg in at least two successive measurements or the detection of a single diastolic pressure ≥ 110 mmHg. The National Collaborating Centre for Women’s and Children’s Health (NCC-WCH), in the guideline (LG) “Hypertension in pregnancy”, defines the following conditions:

- Chronic hypertension in pregnancy: arterial hypertension detected during the first pregnancy check or occurred before the 20th week of gestation.
- Gestational hypertension: new onset of arterial hypertension after the 20th week of gestation, in the absence of proteinuria.
- Pre-eclampsia: new onset of arterial hypertension after the 20th week of gestation, in association with one or more of the following conditions:
  ○ Proteinuria: proteinuria/creatinuria ratio ≥ 0.3 mg/g (30 mg/mmol), persistent dipstick ≥ 3 g/L (+++), or proteinuria 24 h > 300 mg;
  ○ Renal function contraction: serum creatinine ≥ 1.1 mg/dL;
  ○ Signs of liver distress: AST ≥ 50 IU/L and/or severe hypogastric or upper right quadrant pain;
  ○ Neurological disorders: severe headache with hyperreflexia, hyperreflexia with clone, until convulsions (eclampsia);
  ○ Hematological disorders: thrombocythemia, hemolysis.
- Severe pre-eclampsia: if it meets at least one of the following criteria:
  ○ High pressure values characterized by Systolic Arterial Pressure ≥ 160 mmHg and/or Diastolic Arterial Pressure ≥ 110 mmHg in at least two detections at least 6 h apart;
  ○ Neurological disorders (cerebrovascular events, convulsions) or visual disorders (loss of vision);
  ○ Pulmonary edema;
  ○ Epigastric pain in the right upper quadrant;
  ○ Hepatic cytolysis (increase in transaminases by at least two-fold compared to normal values);
  ○ LDH values > 600 IU/L;
  ○ Thrombocytopenia with platelet count < 100,000/mm^3^.
- Eclampsia: appearance of convulsions associated with PE.
- HELLP syndrome: evidence of hemolysis, elevated liver enzymes, reduction in platelet count.

Even though the exact causes and pathophysiology of PE are still unknown, it is thought that the disease develops in two stages. An initial stage involves a placentation deficiency, most likely brought on by an aberrant differentiation of cytotrophoblast cells during their invasion of the spiral uterine arteries, and then a maladjustment of the maternal local immune response to fetal tissues [6,7].

### 1.2. Extracellular Vesicles (EVs)

Among various damaging factors released by the placenta, EVs could play a fundamental role in the onset of hypertensive disorders [8]. Hence, in pregnancy, crucial events such as the migration/invasion of trophoblasts, as well as cellular adaptations to changes underlying gestation, may involve the release of EVs as key modulators [9,10,11].

The International Society for Extracellular Vesicles (ISEV) proposed the term “extracellular vesicles” to define vesicles released by cells [12].

**Figure 1:**
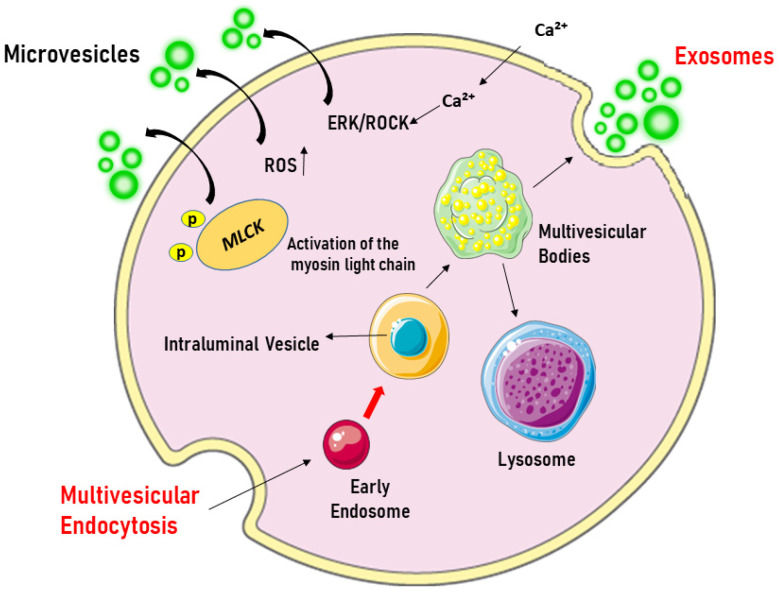
Origin of extracellular vesicles. Microvesicles are formed by blotting the plasma membrane upon cell activation, which triggers the influx of Ca^2+^, leading to phosphatidylserine exposure and the activation of the ERK/ROCK pathways. This is followed by the phosphorylation and activation of the myosin light chain by myosin light-chain kinase (MLCK), which triggers the release of microvesicles. In contrast, exosomes originate from the endosomal compartment, where intraluminal vesicles are formed through the inward growth of early endosomal membranes. As early endosomes mature into multivesicular bodies, they accumulate intraluminal vesicles, which can fuse with lysosomes for degradation or with the plasma membrane to release exosomes into the extracellular space. ERK/ROCK = extracellular signal-regulated kinases/Rho kinase. Created through BioRender.

On the basis of the biogenetic pathway and origin, physical characteristics, and composition, the “extracellular vesicles” can be divided into three distinct types:

(1) Exosomes, with a diameter between 30 and 120 nm, which are released from endosomes as a consequence of the invagination of the endosomal membranes and the formation of intraluminal vesicles within the multivesicular bodies.
(2) Apoptotic bodies, which are formed during apoptosis and have a diameter ranging from 50 to 5000 nm.
(3) Microvesicles (MVs), larger than exosomes (40–1000 nm), which are formed by the outward growth of the plasma membrane due to the involvement of cytoskeleton proteins. Figure 1 represents the biogenesis of EVs.

Most bodily fluids, such as blood, urine, saliva, milk, uterine fluid, cerebrospinal fluid, and synovial fluid, include EVs, which are released by a variety of cell types [13,14,15,16]. It is now widely accepted that EVs represent a critical method for intercellular communication, highly necessary for the survival and maintenance of almost all multicellular systems. They can induce their effects on target cells by merging with the plasma membrane and delivering their content into the cytoplasm, through endocytosis, or through the release of their content into the extracellular space [17]. Regarding cargo, EVs can transport proteins, second messengers, lipids, nucleic acids, and microRNAs from cell to cell and, thus, can mediate the crosstalk between different tissues [18,19,20].

These vesicles are also involved in the elimination of harmful intracellular or membrane-bound components, thus mediating protective effects in intercellular exchanges [21,22].

Since EVs show tissue-specific features and reflect the functional status of the original cells [23], the aim of this review is to analyze their implications in gestational hypertensive disorders and pre-eclampsia and the possible role as indicators of cell function and biomarkers of disease.

## 2. Materials and Methods

A literature search was conducted on the PubMed, Medline, Google Scholar, Scopus, and Web of Science databases using MESH terms such as “Preeclampsia”, “Pregnancy”, “Hypertension”, “Pregnancy-related hypertension”, “Extracellular vesicles”, “Biomarkers”, “Gestation” AND “Obstetrics” and NOT “Environmental Biomarkers” OR “Pediatrics”, without any geographical restrictions. Using those selection criteria, we found 21,200 research products. Then, we restricted the literature search to products published from 1989 to 2025. Book chapters, case reports, (conference) abstracts, non-English publications, and duplicates were excluded, so that 21,092 research products were removed and only full-text original research studies, review articles, and systematic review reports published in peer-reviewed journals were analyzed [24]. Finally, considering in addition otherwise relevant papers, we included 173 research products for writing the review.

## 3. Results

### 3.1. EVs in the Regulation of Physiological Pregnancy

Although information on the role of EVs in the regulation of reproductive physiology and in the pathophysiology of pregnancy-related diseases is scarce, it is fairly well accepted that many of the processes known to involve EVs, such as angiogenesis, cell proliferation, inflammation, and immune response, can be critical for implantation, placental development, and placental function and are therefore essential for the maintenance of the pregnancy [25,26,27,28,29,30,31,32,33,34,35,36,37,38,39,40,41].

Among EVs, MVs and exosomes particularly have been specifically indicated as the main vesicles involved in fetal–maternal communication during implantation and placentation [42,43].

Although the precise origin and functions of EVs in maternal circulation are not well understood, it is widely accepted that the placenta, umbilical cord, and amniotic membranes can secrete EVs as early as 6 gestational weeks in response to hypoxic conditions [10,44,45]. EVs released from the placenta enter the maternal bloodstream primarily through syncytiotrophoblasts (STBs) and express the enzyme placental alkaline phosphatase as a surface marker [46].

Furthermore, their biological functions and composition exhibit changes throughout pregnancy in connection to placental circumstances or pregnancy-related complications, including hypertensive illnesses like pre-eclampsia (PE) and gestational diabetes [47,48,49,50]. For instance, large concentrations of immunosuppressive proteins, including interleukin-10 and transforming growth factor beta 1, are indicative of EVs released during the second trimester of pregnancy [51]. In terms of biological activity, MVs generated by STBs during the same gestational period can increase the expression of interleukin-6 and tumour necrosis factor (TNF)-α in B cells and monocytes while also modifying the expression of cytokines implicated in type 2 immunity [52].

As previously reported, EVs can regulate fetal–maternal communication during implantation and placentation by maintaining cellular metabolic homeostasis, promoting fetal vasculogenesis and growth and maternal uterine vascular adaptation. Thus, they may support gestational vascular changes during pregnancy and prepare the uterus for delivery [53,54]. Their characteristics as cargo transfer facilitators make the aforementioned effects possible. In fact, like other EVs, placenta-derived EVs are abundant in miRNAs, which can regulate the post-transcriptional events and exert their effects by targeting multiple mRNAs [41,55,56]. In turn, miRNAs carried by EVs can influence the proliferation, differentiation, migration, invasion, and apoptosis of trophoblast cells, the function of endothelial cells, angiogenesis, placentation, oxidative stress, and other biological processes. Among placenta-associated miRNAs, 46 miRNAs belonging to the chromosome 19miRNA cluster (C19MC) have been identified in villous trophoblasts [57,58]. The expression of some of the most important C19MC miRNAs changes throughout pregnancy and can be modulated by stress, alterations in circulating glucose levels, and other stimuli. For instance, in trophoblast cells cultured in hypoxic conditions, the expression of miR-1273d, miR-4492, and miR-4417, which are involved in the regulation of immune and inflammatory processes by affecting the human leukocyte antigen-G, was found to be increased [59]. Regarding the effects of the C19MC miRNAs contained in EVs, the ability to cause the proliferation and inhibit the migration of trophoblast cells exerted by miR-519d could be described [60]. Additionally, in patients affected by PE, the fragments of 5′-transport RNA (tRF), which constitute the majority of small RNAs in EVs from STB in normotensive and hypertensive pregnancies, could induce an inflammatory response in tissue macrophages, reduce endothelial nitric oxide synthase (eNOS) expression in endothelial cells, and contribute to the onset of PE [61]. Furthermore, miR5165p, miR5173p, miR5185p, miR2223p, and miR165p expression was found to be increased across gestation in healthy pregnant women, apart from miR-516-5p, which was not expressed in the second trimester. Differently, in women with gestational diabetes mellitus, serum miR-516-5p was found to be overexpressed, whereas that of miR-222-3p was found to be reduced, in the second/third trimester [62].

The mechanisms through which those EV-related miRNAs would induce gestational diabetes were hypothesized to be related to the modulation of genes involved in the regulation of the insulin resistance pathway.

Finally, placental exosome-derived miR 499 would play a role in pregnancy by downregulating the nuclear factor kappa-light-chain-enhancer of activated B cells (NF-κB) exerted by targeting the Lin28B/let-7-ras signalling axis, and, therefore maintaining a decreased pro-inflammatory profile [63].

### 3.2. Role of EVs in Vascular Remodelling Throughout Pregnancy

Following the occurrence of implantation, decidualization, and placentation, the placenta guarantees the supply of nutrients for optimal embryo development [64]. In this regard, the uterine spiral arteries, which undergo transformations under the influence of adaptive mechanisms, provide the mandatory resources [65]. In particular, the cellular and extracellular components of the uterine spiral arteries undergo changes such as apoptosis, hyperplasia, migration, and remodelling of the extracellular matrix, which are coordinated by cytotrophoblast cells and decidual natural killer (NK) cells [66].

It should be noted that although during pregnancy EVs involved in the regulation of angiogenesis could originate from maternal circulation and cord blood, the activity of maternal serum-derived EVs would be higher than that of those originating from cord blood [67]. This could be related to the differential expression of miRNAs, such as miRNA-550a-5p, miRNA122-5p, miRNA-210-3p, miR376c-3p, miRNA-151a-5p, and miRNA-296-5p, in EVs from the maternal or blood cord [68]. Regarding the possible mechanisms based on the angiogenetic effects of EVs, there could be the release of pro-angiogenic factors, which could enable the normal gestational development and keeping of endothelial function [69]. Additionally, both vascular smooth muscle cells (VSMCs) and endothelial cells could represent targets for EVs. During placental development, VSMC migration was reported to be involved in spiral artery remodelling by mechanisms partly promoted by EVs released by extravillous trophoblast (EVT) cells and the activation of a specific EVT-VSMC exosome communication pathway [70], the transfer of exosome miRNAs, and the release of VEGFA [71]. Moreover, based on the angiogenetic effects of EVs, there would also be the actions elicited by EVT-derived EVs on endothelial cells. In this regard, it was reported that maternal and umbilical exosomes have been reported to be capable of stimulating the proliferation and migration of human vein endothelial cells (HUVECs), as well as angiogenesis [68].

There is also evidence that STB-released EVs contain the eNOS isoform, which plays a pivotal role in the constitutive release of one of the most important vasodilators and regulators of endothelial function, namely nitric oxide (NO) [72]. In this regard, it is worth noting that miR-155, which has been found in placental EVs, can inhibit the eNOS and thus cause endothelial dysfunction [73].

Another source of EVs during pregnancy is represented by human umbilical cord mesenchymal stem cells (hUCMSCs), which have pro-angiogenic activity [74,75]. It was hypothesized that under hypoxic conditions, hUMSC-derived exosomes would be released and that they could induce their effects through pro-angiogenic factors, including VEGF, VEGFR-2, MPC-1, angiogenin, tie-2/TEK, and IGF [76]. Furthermore, the upregulation of miRNA-150, which was associated with the increased expression of VEGF and Notch1, was found in exosomes in animal models of pregnancy. Thus, exosome-derived miRNA-150 could represent a major regulator of angiogenesis during pregnancy. Among others, miR486-1-5p and miR486-2-5p could also be involved in migration, placental development, and angiogenesis [77].

### 3.3. Role of Immunomodulatory Effects of EVs in Pregnancy

During pregnancy, placental EVs can act both as immunosuppressive and immunostimulatory factors, depending on their cellular origin and cargo [78]. For example, EVs released from the STB and containing the placental Fas ligand and CD274 would induce immunosuppression by the inhibition of T cells [79,80]. Instead, EVs containing TNSF10 would act as immunomodulatory factors and promote fetal tolerance, through T cell apoptosis, and the prevention of the degradation of trophoblast cells [79]. Furthermore, EVs expressing NKG2D ligands, MHC class 1 chain-related molecules A and B, and six cytomegalovirus UL 16-binding proteins were found to inhibit NK cells by the activation of NKG2D receptors [81,82]. Finally, placental EVs may also negatively regulate T cells and prevent the overstimulation of the immune systems via the CD3-zeta and Jak3 pathway [80]. Studies of viral resistance in pregnancy have demonstrated that EVs’ transport of miRNAs may also play a role in maternal immune modulation. It was shown that at least three members of the C19MC miRNAs family—mir517-3p, mir516B-5p, and mir512-3p—have potent antiviral properties, and that their expression in primary human trophoblast-related EVs can lower viral infection in non-placental cells.

The results of those studies showed that, on the basis of maternal immune modulation and protection against viral infection, there would be mechanisms related to autophagy [82,83]. Furthermore, miR-517a-3p, which was found to be highly expressed in trophoblast-derived EVs, would regulate T cell activation and NK cells via the NO pathway [84]. In the same way, miR-141, which is contained in the STB-EV cargo, could be involved in immunomodulation through the suppression of T cells [85]. EVs may have pro-inflammatory mediatory effects in addition to the immunosuppressive ones. Indeed, it has been demonstrated that trophoblast cells generate EVs containing fibronectin, which binds to α5β1 to attract monocytes and macrophages [86]. During pregnancy, macrophages are abundant in the decidua and would modulate the inflammatory response by producing cytokines and chemokines. Therefore, EV-associated fibronectin would result in the increased release of pro-inflammatory cytokines, such as interleukin 1β, interleukin 6, serpine-1, and TNF-α [87]. STB-derived exosomes could induce inflammation by stimulating leukocytes and monocytes to increase the expression of pro-inflammatory cytokines [48,88].

### 3.4. Vascular Complications of Pregnancy

As previously reported, gestational hypertension and PE/toxemia are clinical conditions characterized by high maternal morbidity and fetal mortality [39]. The features of PE are poor placentation and endothelial dysfunction, which would result in multi-organ failure because of arterial hypertension [89]. Impaired maternal endothelial function and uteroplacental circulation are caused by changes in angiogenesis, the elevated production of inflammatory cytokines, and oxidative stress, all of which are characteristics of PE [39,90,91]. Increased maternal inflammatory response and vascular dysfunction would hinder the progression of the placentation, proliferation, invasion, and apoptosis of trophoblasts [92].

In pathological conditions associated with pregnancy, such as gestational hypertension and PE, a link with the placental release of EVs has been highlighted [93,94]. Although the data on the level of circulating EVs in hypertensive disorders are not consistent, being described as both the absence of difference and the increased levels of EVs in comparison with those circulating in normal healthy pregnancies [95,96,97,98], it is quite well established that EVs in gestational vascular disease would contain more pro-inflammatory cytokines and more pro- and anti-angiogenic proteins [99,100,101]. Furthermore, EVs isolated from women with gestational vascular disease would be able to mediate harmful effects in cellular models. In the study by Shomer et al., it was found that exposure of HUVECs to EVs from normal healthy pregnancies could induce stable endothelial tube formation, which could not be observed when EVs from women with gestational vascular disease were used [100].

Additionally, the survival of early-stage trophoblast cells increased when exposed to healthy pregnancy EVs only, by mechanisms related to the extracellular signal-regulated kinases 1/2 pathway, suggesting a role for these EVs in the maintenance of endothelial and trophoblast function [39].

### 3.5. Role of EVs in Gestational Hypertension

After 20 weeks of pregnancy, one of the most prevalent issues for pregnant women is gestational hypertension, also known as pregnancy-induced hypertension, which is characterized by the absence of proteins in the urine. Up to 10% of pregnant women worldwide are afflicted by this illness. Pregnancy-induced hypertension (GHT), chronic hypertension, and PE are the three forms of hypertension that can occur during pregnancy. When GHT occurs, hypertension appears in the latter stages of pregnancy without any further PE clinical signs and goes away after birth [102]. Although the pathophysiology of GHT is still elusive, it is believed to be related to the altered invasion of the trophoblasts and spiral artery remodelling. This would result in a reduction in maternal blood flow to the placenta, the activation of inflammation and platelet aggregation, the damage of trophoblast cells, and endothelial dysfunction. An increase in the release of dangerous substances, like cell fragments and EVs, into the mother’s blood would result from all of the aforementioned circumstances [103]. Total peripheral blood exosomes were reported to increase in pregnant women with hypertensive disorders, which was correlated with the severity of the disease compared to normal pregnancies. Furthermore, umbilical cord plasma-derived exosomes from PE women were reported to be able to cause vascular dysfunction by targeting 3-hydroxy-3-methylglutaryl-CoA synthase in endothelial cells [104].

The pathogenic mechanisms of GHT could also be associated with their content, which is different, especially with respect to miRNAs, from that found in EVs from normal pregnancies. With respect to this issue, exosome-related hsa-miR-210 may play a role in the pathophysiology of GHT [105]. Additionally, the maternal plasma exosome profiling of selected chromosome 19 miRNA cluster microRNAs showed the downregulation of miR-517-5p, miR520a-5p, and miR-525-5p in patients with a later appearance of GHT [106]. Finally, in other studies, miR-516-5p, miR-517, miR-520 h, miR525, miR-526a, and miR-518b were found to be upregulated and associated with the later development of GHT [10,107].

### 3.6. Role of EVs in PE

PE is defined as proteinuria (0.3 g protein in 24 h urine collection) and blood pressure of around 140/90 mmHg or higher after 20 weeks of pregnancy in women who had previously had normal blood pressure [10,108,109]. Symptoms of pre-eclampsia might include swelling of the hands or face, intense headaches, changes in eyesight, breathing difficulties, rapid weight gain, and shoulder or abdominal pain. Women may also have nausea or vomiting in the second half of their pregnancy [110,111,112,113]. The illness is associated with high rates of maternal and fetal death. It is estimated that 76,000 women and 500,000 newborns die from PE each year, respectively, because of these illnesses. Additionally, the newborn may face a raised risk of both acute and chronic illnesses during infancy, childhood, and adulthood [10].

As previously reported, at the base of PE, there would be abnormal placentation caused by altered spiral artery remodelling related to the poor invasion of trophoblast cells. Placentation begins on the 10th day of gestation with the development of trophoblast cells, which invade the endometrial stroma, determining the beginning of the decidual reaction. By the 8th–9th week of gestation, the formation of villi is complete, and, starting from the 12th week, the decidua completely covers the maternal spirals arterioles.

During implantation, placental trophoblasts cause a remodelling of spiral arteries, which is also related to the changing of the phenotype of the trophoblast cells, which acquire an endothelial phenotype and express surface adhesion molecules.

Therefore, the most accredited theories related to the pathophysiology of PE would rely on defects in the remodelling of the placental spiral arteries during the invasion of the trophoblasts of the uterine wall, which would prevent the transformation of the placental uterus circulation into a low-resistance one. Consequently, the perfusion of the intervillous space would be altered and this would be followed by placental hypoxia, ischemic damage, and the release of antiangiogenic and vasoconstrictive factors and pro-inflammatory cytokines [108,114]. According to other theories, PE would not be associated with abnormal placentation but with pre-existing endothelial dysfunction due to oxidative stress and/or pregestational metabolic alterations (e.g., hypertension, obesity, diabetes). Thus, an inadequate adaptive capacity to face the physiological stress induced by pregnancy would favour the onset of microvascular damage [115].

Since the pathophysiology of PE is not well understood, there is no helpful treatment to prevent the long-term consequences of PE [116,117,118,119]. Furthermore, although Doppler velocimetry, maternal factors, and proteinuria could be taken as reliable methods for monitoring PE monitoring [5,120], they cannot be used in the early diagnosis of PE.

Among other possible markers potentially useful for this purpose, EVs could represent good candidates. The first evidence of changes in the circulating levels in PE patients was reported in the study by Knight et al. [111], who demonstrated that plasma EV levels released by STBs increased in the third trimester in patients with PE compared to healthy controls. Since then, in many other studies, the concentration of plasma exosomes of women with PE was reported to be higher, compared to that observed in women without PE, in particular in the third trimester [121,122,123]. For example, Tan et al. and Jadli et al. found higher levels of Annexin V-positive EVs in the plasma of PE women compared to what was found in non-PE women [124,125]. Also, Salomon et al. found plasma EVs in plasma of PE women to be increased compared to healthy subjects. Those EVs were reported to be associated with abnormal levels of soluble fms-like tyrosine kinase-1 (sFlt-1), soluble endoglin (Eng), and placental growth factor (PlGF) and were shown to be capable of activating endothelium, platelets, and monocytes [126,127].

Moreover, the podocin +/nephrin + EVs ratio in the urine of women with PE and the concentration of EVs loaded with sFlt-1 from the saliva and gingival crevicular fluid of PE women were reported to be higher than in non-PE women [128,129,130].

It should be noted that the mean diameter of exosomes derived from pre-eclamptic women was larger than that of exosomes isolated from healthy controls, too [55]. It should be emphasized, therefore, that while most of the evidence points to an underlying increase in EVs in PE, other reports show the contrary. These disparities and contradictory results may be explained by various study methods, EV kinds and sources, PE subtypes, etc. [23].

Changes in EVs concentrations were observed as well as the protein content of EVs in pregnant women with PE, as a response to the hypoxic condition caused by abnormal placentation. For instance, 25 different proteins such as annexins, integrins, histones, heat shock proteins, complement regulation proteins, and various enzymes were identified in EVs associated with PE [131]. Modifications to the EV protein cargo could be involved in the pathophysiology of PE. Therefore, as also mentioned above, Eng and Flt-1, as well as their soluble forms (sEng and sFlt-1), have been reported to be elevated in placental exosomes and MVs of women with PE [42,129,132]. Regarding the possible mechanisms of action of the onset of PE, it could be hypothesized that these factors could reduce the bioavailability of VEGF and placental growth factor, thus promoting vasoconstriction and endothelial dysfunction [133,134]. Furthermore, in human umbilical vein endothelial cells, exosomes derived from patients with PE, containing sFlt1 and sEng, were able to reduce proliferation, migration, and tube formation [133].

Additionally, the development of endothelial vascular dysfunction and, consequently, arterial hypertension may be linked to the genesis of PE through alterations in the amount of eNOS in EVs and, consequently, NO release. Regarding this issue, placental-derived EVs from PE patients showed a decrease in eNOS [72].

The capacity of EVs to induce pro-inflammatory and pro-coagulatory states, as well as trophoblast dysfunction [135], serving as carriers and communication channels between cells, may be another way through which they contribute to the pathogenesis of PE. Regardless of gestational age, exosome miR-486-1-5p and miR-486-2-5p significantly increased in PE in relation to this issue [127].

Similarly, an analysis of miRNAs from the plasma of women who later developed PE evidenced an increase in miR-155, miR-153-3p, miR-325-3p, and miR-342-3p and a decrease in miR-26b-5p, miR-7-5p, and miR-181a-5p [55,73,136,137,138].

Additionally, miR-136, miR-494, and miR-495 were reported to be markedly elevated in exosomes isolated from blood taken in PE patients compared to that found in normal pregnancies [139]. On the contrary, miR-548c-5p was found to be reduced in serum exosomes from patients with PE. Regarding the mechanisms through which these miRNAs could be involved in the onset of PE, there would be effects on eNOS expression and the production of NO in HUVECs [73], the modulation of macrophage proliferation and activation [139], the regulation of tube formation and VEGFA release in endothelial cells, and the modulation of angiogenesis [140,141,142,143].

The existence of 30 miRNAs linked to late-onset PE and 59 miRNAs associated with early-onset PE was demonstrated in a study on the differential expression of exosome miRNAs in pregnant women with early-onset and late-onset PE. The deregulation of several biological processes, such as cell migration and invasion, cell proliferation, apoptosis, mesenchymal transition, and angiogenesis, was linked to the elevated miRNAs. Rather, two miRNAs—miR-2113 and miR-374c-5p—which were downregulated in patients with early- and late-onset PE—were connected to inflammation and lipid metabolism [23].

According to the literature, there is an increase in different types of EVs in the biological fluids of pregnant women in cases of PE. Changes in the proteomic profile and miRNA of EVs could represent the underlying cause of alterations in trophoblast function, angiogenesis, inflammation, and endothelial dysfunction in PE.

However, it should be highlighted that the complex interactions of maternal cell-derived EVs and placental-derived EVs should be better addressed to clarify the mechanisms of initiation and progression of PE. An improvement of knowledge about quantitative alterations in EVs, the content of bioactive molecules (mRNA, miRNA, proteins, lipids, and metabolites), their cellular origin, and their interaction with target cells may be useful in identifying the underlying mechanisms that contribute to the pathophysiology of PE.

The literature indicates that pregnant women with PE have higher levels of several EV types in their bodily fluids. The underlying cause of changes in trophoblast function, angiogenesis, inflammation, and endothelial dysfunction in PE may be changes in the proteomic profile and miR-NA of EVs. It should be noted, therefore, that to better understand the mechanisms behind the onset and progression of PE, the intricate interactions between EVs produced from maternal cells and placental cells need to be properly addressed. Finding the fundamental processes that underlie the pathophysiology of PE may be aided by a better understanding of the quantitative changes in EVs, the composition of bioactive molecules (mRNA, miRNA, proteins, lipids, and metabolites), their cellular origin, and their interactions with target cells. A deepening of the understanding of the roles of EVs in the pathogenesis of PE may allow the development of a panel of biomarkers, which could be useful for identifying pregnant women at risk of developing PE.

As the maternal vascular, immune, and coagulation systems may have bidirectional communication, it could be possible to use EVs as a therapeutic tool to target one component of the maternal system, in order to facilitate the other two systems to return to the physiological condition [115].

**Figure 2:**
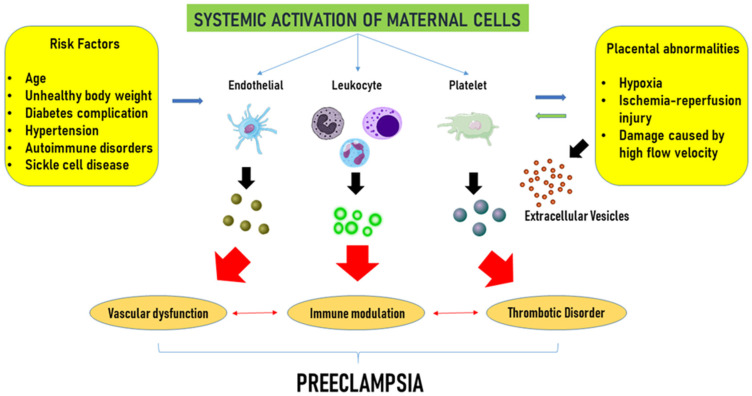
Extracellular vesicle-related complications in pre-eclampsia. Advanced maternal age, overweight, hypertension, diabetes, and autoimmune disorders, widely considered maternal risk factors, and placental abnormalities, such as hypoxia and ischemic injury, trigger the activation of maternal endothelial, platelets, and leukocytes, which in turn release extracellular vesicles. Extracellular vesicles would contribute to vascular dysfunction, immune modulation, and an increased risk of thrombotic complications, collectively advancing the progression of the disease. Modified from Gilani et al. [115] Curr. Hypertens. Rep. 2016, 18, 68, doi:10.1007/s11906-016-0678-x. This article is Open Access and distributed under the terms of the Creative Commons Attribution 4.0 International License.

The role of EVs in the genesis of pregnancy-related hypertension and PE is depicted in Figure 2.

### 3.7. Protective Effects of EVs in Pregnancy-Related Hypertensive Disorders

In addition to their role in pathophysiology, EVs also emerge as therapeutic agents in regenerative medicine and as targeted drug delivery [144]. The use of EVs as a therapeutic tool could have higher biocompatibility and lower immunogenicity and should be easier than cell therapy. However, translating EV-based therapeutics into clinical scenarios presents multifaceted challenges, which range from technical to regulatory compliance issues [145].

Concerning pregnancy and pregnancy-related hypertensive disorders, EVs, in addition to exerting harmful actions and representing potential mechanisms for the onset of GHT and PE, could also act as protective mediators able to antagonize anti-angiogenic factors and vascular injury. Indeed, a large number and type of EVs have been reported to promote angiogenesis, such as those released from endothelial cells, platelets, and, particularly, from mesenchymal stem cells (MSCs) [146,147]. In fact, current clinical trials aimed at evaluating the therapeutic potential of EVs [145]. In relation to this issue, it was reported that human umbilical mesenchymal stem cell (HUMSC)-derived EVs can significantly activate the migration, proliferation, and tube formation of HUVECs, as well as increase the expression of the pro-angiogenic factor VEGF [148,149].

Targeting prediction analysis showed that various EV miRNAs are related to the regulation of tissue repair, and five miRNAs can directly target VEGF-A. Among others, miR-17-5p was found to promote endothelial cell proliferation, migration, and tube formation by targeting the PTEN/AKT/HIF-1α/VEGF pathway [147,148,149,150].

Also, human adipose stem cell-derived EVs could exert protective effects on angiogenesis through the let-7/argonaute 1 (AGO1)/VEGF pathway [150].

In addition to acting as pro-angiogenetic factors by increasing VEGF production, EVs could also reduce its degradation or increase the expression of VEGF receptors. Regarding the first issue, Q. Li et al. [151] found that the expression of miR-486-5p was increased in stem cell-derived EVs in response to hypoxia. Furthermore, miR-486-5p was able to reduce VEGF degradation and promote angiogenesis in animal models of myocardial ischemia.

Other possible mechanisms through which EVs could improve angiogenesis could be related to the modulation of the PI3K/Akt/eNOS pathway [152,153,154], autophagy [155], and osteocalcin release [156].

It should be noted that EV therapy could alleviate the symptoms of PE by reducing endothelial cell inflammation as well. About this issue, macrophage-derived EVs were found to reduce the expression of TNF-α and interleukin 6 and inhibit the inflammatory response of endothelial cells [157]. In other studies, EVs derived from mesenchymal stem cells have been reported to counteract endothelial cell inflammation by downregulating the toll of receptor 4, NF-κB p65 subunit phosphorylation, and caspase-1 expression [158]. miR-18b in HUMSC-derived EVs seems to be able to exert anti-inflammatory effects by inhibiting the MAP3K1/NF-κB axis [159].

Similarly, Li et al. [160] observed that miR-17–3p in HUMSC-derived EVs could reduce inflammatory factors and markers of oxidative stress and increase the activity of superoxide dismutase and glutathione peroxidase in rats. Finally, Zheng et al. [161] showed that decidual mesenchymal stem cell-derived EVs could improve serum-induced endothelial dysfunction, thereby reducing the level of inflammatory factors and lipid peroxidation, promoting cell proliferation, and counteracting inflammation and oxidative stress in cellular models of PE and in patients with PE.

The protective effects elicited by EVs in pregnancy-related hypertensive disorders could also be related to immunomodulatory effects and actions on trophoblast function. In many studies, trophoblasts were shown to act as a possible target for eliciting the protective effects of EVs in hypertensive disorders associated with pregnancy.

The administration of EVs in a heme oxygenase-1 knockout mouse model of PE was able to improve miscarriage rates, fetal growth restrictions, uterine artery remodelling, and maternal PE symptoms. These findings were related to the increase in CD103-positive cells associated with the induction of immune tolerance, the reduced expression of interleukin 6 and interferon γ, the increased expression of interleukin 10, and the regulation of immunity [162].

Li et al. [163] showed that EVs released by mesenchymal stem cells derived from chorionic villus-derived mesenchymal stem cells caused trophoblast migration and proliferation by increasing the expression of TRIM72, which was followed by p53 ubiquitination, proteasomal degradation, and the inhibition of apoptosis. miR-100-5p in endometrial epithelial cell-derived EVs could increase trophoblast proliferation, invasion, migration, and angiogenesis. Others [164,165,166,167,168] observed similar effects regarding the role of miR-133b, miR-18b, miR-139-5p, and miR-101 in EVs from human umbilical cord mesenchymal stem cells (hUC-MSCs).

**Figure 3:**
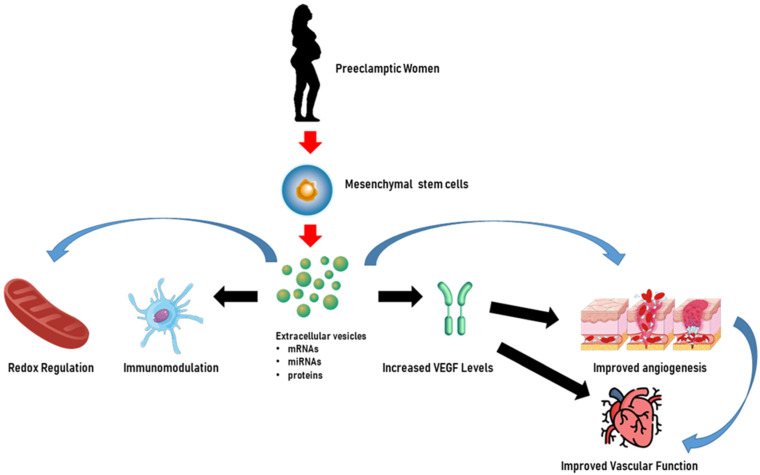
Extracellular vesicles as potential therapeutic tools for the treatment of pre-eclampsia. In pre-eclamptic women, extracellular vesicles derived from human umbilical mesenchymal stem cells may enhance endothelial cell function by increasing vascular endothelial growth factor (VEGF) levels and angiogenesis. Furthermore, extracellular vesicles can mitigate inflammation and oxidative stress and exert immune modulation, which are critical in the treatment of pregnancy-related hypertensive disorders, showcasing their potential therapeutic importance. The content of extracellular vesicles, which could be represented by miRNAs, mRNAs, and proteins, could be involved in all the above mechanisms. Created through BioRender.

In addition to miRNAs, Chen et al. [169] evaluated the effects on trophoblasts of long non-coding RNA (lncRNA) H19 in EVs released from bone marrow mesenchymal stem cells and showed that H19 was capable of causing trophoblast migration and invasion and the inhibition of apoptosis by antagonizing miRNA let-7b, increasing FOXO1 expression, and activating the AKT pathway. Figure 3 illustrates the beneficial role of HUMSC-derived EVs in pre-eclamptic women.

In Table 1 and Table 2, a summary of the role of EVs in the genesis of pregnancy-related hypertension and as a therapeutic tool for the management of pregnancy-related hypertension and PE, is shown.

## 4. Discussion

Pregnancy-related hypertensive disorders remain a significant concern in modern obstetrics and gynecology, and they account for high percentages of maternal and child morbidity and mortality [23]. For this reason, it would be highly recommended to identify the population of pregnant women at risk of these conditions.

Despite recent advances in the field, knowledge about the pathophysiology of the aforementioned disorders is still poor and, for this reason, accurate diagnostic methods are lacking [170]. The presence of one or more risk markers during pregnancy determines whether a patient is at low or increased risk of PE. One potential assessment tool is screening using blood biomarkers and Doppler ultrasonography velocimetry of the uteroplacental circulation. To reduce early types of PE, preventive measures must begin before 16 weeks of pregnancy, when most of the physiological transition of uterine spiral arteries takes place. Even with these resources, we still need to examine the etiological mechanisms behind these illnesses in greater detail and take into account a number of factors, such as EVs [5]. Unfortunately, we are still far from being able to predict and prevent pregnancy-related hypertension or PE. In terms of the latter condition, recommendations, mainly based on anamnestic risk factors, can really lead to the identification of only 30% of women at risk. Recently, other strategies have been used to identify a possible screening test for the onset of hypertensive disorders and PE in pregnant women, but none of them was predictive and only a few have been introduced into clinical practice. This is mainly because, despite the extensive research in the field, the etiology and pathogenesis of pregnancy-related hypertensive disorders remain poorly understood.

According to this viewpoint, EVs have great promise because they are essential to the smooth development of pregnancy and play a role in the development of pregnancy-related complications, placental malfunction, and decreased fetal–maternal communication. Additionally, the primary benefit of employing EVs is their easy extraction from various biological fluids (plasma, serum, urine, saliva, breast milk, amniotic fluid, and others), which minimizes patient discomfort, speeds up analysis, and lowers expenses. EVs may be useful indicators for the early detection of fetal developmental abnormalities and pregnancy-related problems since they can be found in the peripheral blood as early as the first trimester of pregnancy. An observational study conducted in 2017 by Motawi et al. examined the differential expression of miRNA-136, miRNA-494, and miRNA-495 in EVs isolated from the peripheral blood of 100 patients with PE versus 100 patients with normal pregnancies. The results obtained showed that these miRNAs were overexpressed in patients with PE, highlighting the potential of EV cargo as biomarkers for the early identification of women at risk of developing PE [138].

Some studies have also addressed the potential for exosome fetal cell-free DNA, which may trigger maternal excess inflammatory responses, as a biomarker [61]. A recent large cohort of 803 banked plasma samples revealed that EV-bound TIMP-1, PAI-1, and PlGF could improve the predictive robustness of existing PE biomarkers [19]. In women with a history of PE, elevated levels of a group of EV markers, including tissue factor and CD117, may be associated with future coronary artery calcification, reflecting a potential role in the diagnosis of PE [62].

Moreover, the advantages of their use as biomarkers could be underscored by the fact that they are of tissue-specific origin and reflect the functional status of the original cells. Also, the use of EVs could also represent an innovative therapeutic tool for the treatment of hypertensive disorders in pregnancy, minimizing possible complications for newborns.

### Limitations and Future Directions

As previously reported, in recent years, EVs, which may play a role in the onset of pregnancy-related hypertension and PE by acting as intercellular communicators, have emerged as possible good biomarker candidates [170]. However, although there are clinical data indicating a correlation between circulating EV concentrations and the emergence of pregnancy-associated complications, it is premature to employ them as biomarkers. In particular, the absence of standardized methods useful for the detection of EVs in biological fluids and for the analysis of EV content has represented a bias in the optic of achieving a sufficient evidence base for their introduction into clinical scenarios. Indeed, EV characterization is complex, time-consuming, and requires highly skilled specialists, which highlights the need for specific laboratory tools. Also, the existence of high variability in pre-processing blood samples and in EV isolation methods may account for the wide range of reported EV concentrations in the blood of pregnant women. It was demonstrated that following the collection of blood, any delay in the first centrifugation can increase the detection of Annexin-V+ EVs [171]. Furthermore, freezing platelet-containing blood samples and plasma may change the release of EVs [172] compared to fresh samples.

Moreover, various isolation methods can affect the quantification and characterization of EVs. Currently, there is no preferred technique to achieve this purpose, although, among others, differential ultracentrifugation, membrane filtration, and immuno-capture have been highly adopted in recent years. However, it should be considered that the choice of isolation method may have an impact on downstream analysis and the interpretation of results. For this reason, when choosing the method to be used, consideration should be given to the potential advantages and disadvantages of each approach.

Another issue related to the use of EVs, is the nomenclature, as highlighted by the MISEV guidelines and their update [12]. In terms of pregnancy and pregnancy-related disorders, the term STBM, which denotes syncytiotrophoblast microvesicles or microparticles, has been widely used. However, in some studies, STBM was adopted regardless of whether EVs have shown a syncytiotrophoblast derivation. Recently, the term STBM has been replaced by ‘exosome’ and ‘extracellular vesicle’. However, most of the studies that used the term ‘exosome’ did not perform isolation [40].

The last MISEV2024 guidelines gave more information about EVs and outlined key methods for their isolation and analysis [12]. The application of the suggested approaches may be helpful, for instance, in reaching a consensus regarding the concentrations of EVs in different pregnancy-related pathologies and about the diagnostic potential of this parameter.

At this time, the most promising candidates in terms of biomarkers appear to be the miRNAs carried by EVs, since similar results have been obtained in several studies. As demonstrated above, the expression of most miRNAs linked to pregnancy complications undergoes alterations, which can be detectable as early as the first trimester of pregnancy. This would suggest that miRNAs isolated from the blood of pregnant women from the first months of pregnancy may be useful as potential indicators of the likelihood of complications. Such an early diagnosis would facilitate timely intervention, thereby reducing the risk of harm to the woman and the fetus.

It should also be considered that the diagnostic methods based on the use of EVs cannot be generalized in a “one-size-fits-all” manner, since the EVs release and cargo may change from patient to patient due to genetics, lifestyle, sex, and age factors. For this reason, a personalized EVs multi-omics-based diagnostic assay should be developed, and multicentric and prospective cohort studies performed in pregnant women at risk of developing PE will be warranted to validate its relevance in the clinical setting [173].

Also, in terms of the use of EVs as therapeutic tools, the main bias is related to the incomplete mechanistic understanding of the therapeutic effects of EVs, which could be associated with the lack of valuable methods to examine the interactions of EVs in the physiological setting. Furthermore, additional information about appropriate storage, handling, and transportation of EV-based therapeutics will be needed to establish regulatory application documents.

Finally, although clinical trials have not reported any adverse events after EV administration to date, toxicity could be reported as a consequence of the use of different sources of EVs, isolation methods, dosages, and patient populations. In this perspective, the development of EV-based therapeutics could benefit from what was learnt from the approval of cell-based therapies, which faced similar problems in their translation into clinical scenarios. The use of bioengineering strategies aimed at changing EVs and their cargo could face this issue and be useful to scale up EV production [144].

## 5. Conclusions

In view of the above considerations, it appears clear that a deeper knowledge relating to EVs would be mandatory for the improvement of the treatment of patients with pregnant hypertension and PE with undoubtedly positive implications also in terms of economic resources and reductions in healthcare costs, too. Indeed, it should be highlighted that current biomarkers, which are used to examine the evolution of PE during the various stages of pregnancy, have limited accuracy and alignment with the progression of the disease.

To better understand the mechanisms of PE initiation and progression, it is especially important to explore the intricate connections between EVs produced from maternal cells and placental cells. To further understand the underlying mechanisms that contribute to the pathophysiology of PE, more information is needed regarding the quantitative changes in EVs, the content of bioactive molecules (mRNA, miRNA, proteins, lipids, and metabolites), their cellular origin, and their interactions with target cells. Deepening knowledge of the functions of EVs in the pathophysiology of PE could help the setting up of a panel of biomarkers that could be helpful in identifying pregnant women at risk of developing PE.

Our knowledge of the dynamics of EVs during pregnancy and how they alter during disease may be further refined by additional longitudinal research. Novel data may also result from the use of placental markers other than PLAP. The inadequate reporting of approaches, which impedes interpretation and replication, and a mismatch between methodology and language, were the two main problems we found in the field. Future research must address these problems, even if the studies included in this review suggest that EVs represent a potentially promising biomarker for pregnancy-related diseases. These problems might be resolved with the help of the standardization of methods, but this would necessitate different attention on the base of high or low risk for gestational diseases, as well as a consideration of logistical and financial constraints at various study locations.

As a first step, improved methodological rigour and reporting, with help from sources such as MISEV and EV-TRACK, will allow us to determine the potential source of differences in EV concentrations and greatly enhance our ability to identify useful biomarkers for pregnancy pathologies. Since the heterogeneity of EVs is crucial for regulatory approval, the separation and characterization of EV subpopulations could be performed using nanoscale cytometric methods and affinity-based methods, which could improve therapeutic and diagnostic capacity [145].

For the above reasons, the deepening research on EVs will eventually provide researchers and clinicians with novel, sensitive, and ideally non-invasive biomarkers for the prevention and management of PE.

## Figures and Tables

**Table 1:** Role of EVs in the pathophysiology of pregnancy-related hypertension and pre-eclampsia.

EVs	Cargo	Actions	Pathways	References
HUMSC/Wharton’s Jelly Mesenchymal Stromal Cells–Bone marrow, adipose tissue-derived EVs or exosome, MSC-derived EVs/exosomes, Decidual MSC-derived EVs; stromal cell-derived EVs, Chorionic villus MSC-derived EVs, Endometrial cell-derived EVs	miR-101, 8 miR-133b, miR-18b, miR-139–3p, miR-146a-5p/miR548e-5p, H19 miRNA, miR-486–5p, miR-210–3p, miR-17-5p, long non-coding RNA (lncRNA)H19, miR-126, miR-139 p, miR-17–3p, miR-100–5p, let-7 family- miRNA	Trophoblasts: TRIM72/P53, BRD4/NF-κB/CXCL11 SGK1, Notch2/TIM3/ mTORC1, PTEN/ERK/MMP-2 NF-κB, MAPK, let-7/FOXO1/AKT, EZH2/mTOR endothelial cells:VEGF-A/THBS1, PTEN/AKT/hypoxia inducible factor-1α (HIF-1α)/VEGF, Let-7 AGO1/VEGF, MMP19/VEGFA, VEGFR1, VEGFR2/SRC, AKT, ERK, VEGF/sFlt-1, PTEN/AKT/HIF-1α/VEGF, miRNA let-7b, FOXO1 and AKT, MAP3K1/NF-κB, p-Akt/Akt and p-eNOS, caspase 3, EGF, PDGF, and bFGF, Toll like receptor 4, phosphorylation of the NF-κB p65 subunit; Caspase-1 expression, Increased antioxidants (superoxide dismutase and glutathione peroxidase),Reduction interleukin 6, TRIM72-p53 ubiquitination; Proteasomal degradation and inhibition of apoptosis, Let-7/argonaute 1 (AGO1)/VEGF	Pro-proliferation, pro-migration and anti-apoptosis, Anti-inflammatory effects, Autophagy, Angiogenesis, Tube formation, Improvement endothelial dysfunction, anti oxidant effects, Immunomodulation, Trophoblast migration and proliferation	[146,147,148,149,151,152,153,157,158,159,160,161,162,163,164,165,166,167,168]
Stem cell-derived small EVs	miR-486-5p	MMP19-VEGFA	Angiogenesis	[150]
iPSC, iMSC-EVs		Modulation of autophagy through STAT3 pathway	Angiogenesis and tube formation	[154]
Exosomes derived from osteocalcin-overexpressed EPCs		Osteocalcin- G protein-coupled receptor family C group 6 member A (GPRC6A) pathway	Proliferation and migration and tube formation	[155]
Macrophage-derived EVs		TNF-α and interleukin 6	Anti-inflammatory effects	[156]

**Table 2:** Role of EVs as a therapeutic tool in pregnancy-related hypertension and pre-eclampsia.

EVs	Cargo	Actions	Pathways	References
Placental exosomes	miR-223-3p, miR-297, miR-640, miR-378b, miR-26a-5p, miR-153, miR-126-3p, miR-3a-3p, miR-505-3p, miR-374c-5p, miR-324-3p, miR-499a-5p, miR-504-5p, miR-1275, miR-452-5p, miR-150-5p, miR-210, miR-153, miR-486-1-5p, miR-486-2-5p	Angiogenesis Inflammation Cell proliferation and invasion Vasodilation	Modulation of: fused (SuFu), Fus-1, VEGFA expression, H2S pathways, VCAM1 expression; Limitation of leukocyte adherence to endothelial cells; Modulation of autophagy ATG12-mediated; Regulation of chemokine receptor in macrophages; Regulation of MID1 protein expression; Modulation of HIF-1α expression; Modulation of CPEB3/EGFR axis, IGF-1R and CCR7; Modulation of proliferation and apoptosis by targeting the Brg1 gene, LOXL2 and MEF2C; Modulation of the Hedgehog pathway transcription gene GLI3; Downregulating the potassium channel modulatory factor 1; Reduced eNOS activation	[10,101,103,126,142]
Plasma exosomes/EVs	miR-517-5p, miR 520a-5p, miR-525-5p miR-516-5p, miR-517, miR-520 h, miR-526, miR-525, miR-518b, miR-486-1-5p, miR-486-2-5p, soluble fms-like tyrosine kinase-1 (sFlt-1), soluble endoglin (Eng), placental growth factor (PlGF)	Angiogenesis Activation of endothelium, platelets, monocytes; Trophoblast dysfunction; Endothelial cells proliferation, migration, and tube formation		[10,104,126,133]
Umbilical cord plasma-derived exosomes	miR-342-3p, 3-hydroxy-3-methylglutaryl-CoA synthase 1 (HMGCS1)	Endothelial cells dysfunction		[10,102]
Placenta-associated exosomes	miR-155	eNOS inhibition		[72]
Serum exosomes; placental mononuclear cells-derived exosomes	miR-548c-5p, PTPRO/NF-κB axis	Inflammation		[138]
